# High temperature and nib acidification during cacao-controlled fermentation improve cadmium transfer from nibs to testa and the liquor’s flavor

**DOI:** 10.1038/s41598-024-62609-8

**Published:** 2024-05-28

**Authors:** Ivan D. Camargo, Lucero G. Rodriguez-Silva, René Carreño-Olejua, Andrea C. Montenegro, Lucas F. Quintana-Fuentes

**Affiliations:** 1 Corporación Colombiana de Investigación Agropecuaria – AGROSAVIA, La Suiza Research Center, Km 32 Route to Sea, 687527 Santander, Colombia; 2 Corporación Colombiana de Investigación Agropecuaria – AGROSAVIA, Tibaitatá Research Center, Km 14 Route Mosquera-Bogotá, 250047 Cundinamarca, Colombia; 3 Facultad de Ingeniería, Universidad Nacional Abierta y a Distancia, Calle 14 Sur No. 14 – 23 Barrio Restrepo, 111511 Bogotá, Colombia

**Keywords:** Nib cadmium mitigation, Cadmium concentration gradient, Genotype by environment interaction, Sensory analysis, Flavor quality, Non-metrical multidimensional scaling, Plant sciences, Plant physiology

## Abstract

Migration of nib Cd to the testa during fermentation can be achieved with high temperatures (> 45 °C) and low nib pH values (< 5.0) using spontaneous fermentation. However, this low pH can lead to low flavor quality. This study used three controlled temperature fermentation treatments on three cacao genotypes (CCN 51, ICS 95, and TCS 01) to test its effects on the nib pH, the migration of nib Cd to the testa, and the liquor flavor quality. All treatments were effective in reducing the total nib Cd concentration. Nevertheless, the treatment with the higher mean temperature (44.25 °C) and acidification (pH 4.66) reached the highest mean nib Cd reductions throughout fermentation, a 1.37 factor in TCS 01, promoting the development of fine-flavor cocoa sensorial notes. In unfermented beans, the Cd concentration of nibs was higher than that of the testa, and the Cd migration proceeded down the total concentration gradient. However, Cd migration was observed against the concentration gradient (testa Cd > nib Cd) from the fourth day. Cd migration could increase by extensive fermentation until the sixth day in high temperatures and probably by the adsorbent capacity of the testa. Genotype-by-treatment interactions were present for the nib Cd reduction, and a universal percentage of decrease of Cd for each genotype with fermentation cannot be expected. Selecting genotypes with highly adsorbent testa combined with controlled temperatures would help reduce the Cd concentration in the cacao raw material, improving its safety and quality.

## Introduction

Food security has been defined as “when all people, at all times, have physical and economic asses to sufficient safe and nutritious food preferences for a healthy and active life”^1^. To guarantee this, it is important to consider safety, which is the assurance that food will not cause harm to the consumer when it is prepared and (or) eaten according to its intended use^2^. Food safety has become important worldwide due to consumers’ greater demand for quality and increased international trade. Latin America’s population faces many challenges in producing and affording a healthy diet for several crops^3^, including cacao^4^. One of the challenges of cacao farmers from Latin America is that elevated cadmium (Cd, see Table S1 for abbreviations used) concentrations have been found in samples from this origin^5–8^, and potentially might contribute to dietary Cd exposure, which has been associated with human health effects, mainly renal tubular dysfunction, osteomalacia and osteoporosis^9^. The Cd is classified as carcinogenic to humans by the International Agency for Research on Cancer (IARC^10^).

Compared to Africa or Asia, cacao beans from Andean countries have been showing concentrations four to six times higher^11^, with a more considerable variability of Cd concentrations in its young soils ranging between 0.22 and 10.8 mg kg^−1^^11^. These soil Cd concentrations can influence bean Cd concentrations, increasing with total soil Cd and decreasing soil pH and organic carbon^11^. Several studies have reported a mean of bean Cd concentration ranging from 0.10 to 12 mg kg^−1^ for Latin American Countries^11^, including Colombia^8,12^. Although there is no regulation for cacao beans’ cadmium concentration, it is only for derived products; this range of values exceeds both the maximum content of Cd established at 0.8 mg kg^−1^ by the European Commission^13^ and the unofficial industry limits confronted by cacao farmers, reported between 0.50 and 1.10 mg Cd kg^−1^^14^ but as low as 0.10 mg Cd kg^−1^ established by some purchasers^11^.

The impact of spontaneous fermentation as a mitigation strategy for the bean Cd concentration has been formally proposed recently by two studies^15,16^, indicating a potential internal translocation of Cd from nib to testa against the concentration gradient^16^, resulting in a reduction factor (RF) of 1.3 in nib Cd, that is dependent on both, more extensive fermentations than achieve nib pH values lower than five^15^, and temperatures higher than 45 °C^16^. Another study, using incubation-like fermentation, confirmed that higher temperatures may be necessary for nib Cd reduction due to the breakdown of phytate and the release of associated Cd^2+^ ions^17^. Furthermore, it has been suggested that the testa’s ability to absorb heavy metals could contribute to the migration of Cd from nibs to testa^15^. This effect is more pronounced (up to 3.41 times greater than in the nibs) when exposed to high temperatures and acidification^18^. However, this hypothesis needs further validation since the migration of Cd to testa goes against the concentration gradient observed in unfermented beans^15^.

The need for research on the potential impact of controlled temperature conditions allowing acidification-driven mobilization through fermentation has been indicated^16^. However, fermentation with later-stage pH values lower than five has been related to off-flavors^19,20^, suggesting the need for additional research on a balance of the effect of fermentation on Cd reduction and the flavor quality of the final product^15^. Temperature has a significant role in reducing cadmium in beans and improving flavor quality. Studies of spontaneous fermentation have shown that high temperature is instrumental to the death of the seed in the first 48 h and impacts the structural barriers that separate the enzymes and substrates of flavor formation, favoring that acetic acid diffuses into the seeds and acts as a solvent for these cellular materials^21–24^.

Recent research showed that during spontaneous cacao fermentations, two phases of differential metabolic activity correspond with the observed temperature variations: an exothermic and an isothermic phase^25^. A continuous increase in temperature characterizes the exothermic phase until the fourth day of fermentation, dominated by exothermic oxidation reactions that can lead to temperatures reaching 45 °C. On the other hand, the isothermic phase, which starts from day four, is characterized by lower metabolic activity and low fluctuations in temperature, typically around 5 °C^25^. During the exothermic phase, a high temperature between 45 and 50 °C leads to increased diffusion of acetic acid, lactic acid, and ethanol into the nibs, promoting the degradation of flavonoids, which in turn reduces the bitterness and astringency of the cocoa^23,25^.

Other studies have confirmed the preponderant importance of temperature^23,25^, pH^23^ and fermentation time^26,27^ in the sensory quality of the grain using spontaneous or biochemical controlled fermentation. The effect of the expression of genetic variation in response to fermentation has been documented for the sensory quality of cacao by some studies^27–31^. However, to the best of our knowledge, the role of genetic variability in response to fermentation for Cd mitigation remains unexplored. One pioneer study investigating the mitigation of Cd throughout fermentation^15^ has included the variety CCN51 and Nacional from Ecuador. However, the approach to treatment application was not completely randomized, and the inference of genetic variation for the mitigation of Cd is not possible.

Addressing the issue of emulating climatic conditions of spontaneous cacao fermentation using controlled temperature can help to seek its future repeatability in process control strategies of easy implementation and to design systems and tools for sustainable fermentation in small and medium-scale cacao production farms, which allows its traceability, from the point of view of flavor quality^25^, but also its safety in terms of reducing cadmium content.

This study aims to understand the impact of the interaction between controlled temperature conditions and three cacao genotypes on the migration of Cd from nibs to testa throughout fermentation. The questions were:

(i) Is the increase in the mean temperature throughout cacao-controlled fermentation related to a greater decrease and increase of nib and testa pH, respectively?
(ii) Is there a relationship between the increase in temperature and the decrease in nib pH with the translocation of Cd from the nibs to the testa?
(iii) Does a greater decrease in nib pH during fermentation impact flavor quality, and how does temperature affect this process?

## Results

### Fermentation temperature and variation of the nib and testa pH

The average temperature (± s.d.) throughout fermentation of the different treatments were T1: 41.14 ± 3.84 °C; T2: 42.43 ± 4.39 °C and T3: 43.86 ± 4.74 °C. Temperature until the fourth day increased in all treatments, simulating the exothermic phase of fermentation with mean temperatures (± s.d.) of T1: 40.00 ± 4.06 °C; T2: 41.00 ± 4.47 °C and T3: 42.6 ± 5.18 °C. From the fourth day on until the sixth day, temperatures were isothermal with mean temperatures (± s.d.) of T1: 44.00 ± 0.1 °C; T2: 46.00 ± 0.1 °C and T3: 47.00 ± 0.1 °C.

**Figure 1:**
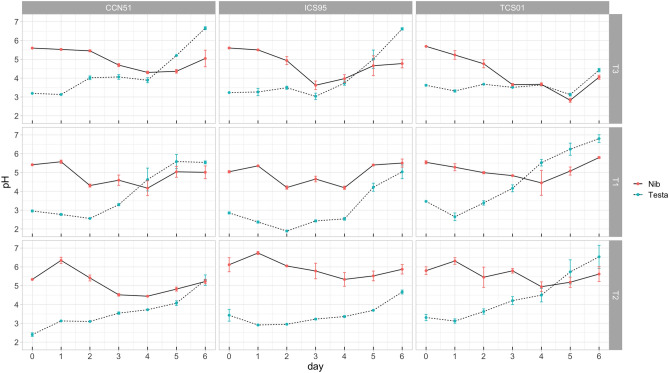
Time course for the pH of cacao bean tissues (nib and testa) of three genotypes (CCN 51, ICS 95, TCS 01) fermented under three temperature profiles (T3, T1, T2) for 6 days. Means (dots) for three sample units and standard errors are plotted.

The significance of the Day factor and the Day by Treatment interaction in Table 1 indicates that the nib pH decreases throughout time (Fig. 1). The higher decrease concerning the value of day zero occurred on the fourth day of the fermentation in most treatments (except T3, fifth day), on average 19.88, 14.61, and 29.84% for T1, T2, and T3, respectively. After the fourth day until the sixth day, genotypes in all treatments increased the pH to a lower extent than the mean value of day zero, except for T1, whose mean value of the sixth day was higher than the mean value of day zero for two of three genotypes (Table S2, Fig. 1, Fig. S1). Treatments were significantly distinct for the mean pH observed throughout fermentation (Table 1), T1: 4.97 (4.81–5.14, 95% C.I.), T2: 5.55 (5.39–5.72, 95% C.I.), T3: 4.66 (4.50–4.83, 95% C.I.). More interestingly, the higher-order interactions for this trait are all significant: Day by Genotype and Day by Genotype by Treatment, respectively, reflecting genetic variation for the nib pH through fermentation in each treatment (Table 1, Table S2).

**Table 1:** Repeated-measures ANOVA for the pH of cacao bean tissues of three genotypes (G) fermented under three temperature profiles (T) for 6 days (D).

Effect	d.f.n	d.f.d	Nib pH	*P*	Testa pH	*P*
T	2	18	12.813	**9.37 × 10**^**–07**^	0.484	0.183
G	2	18	0.674	0.204	7.747	**1.87 × 10**^**–06**^
G × T	4	18	2.836	**0.001**	6.676	**3.03 × 10**^**–07**^
D	6	108	6.573	**2.43 × 10**^**–31**^	27.881	**5.75 × 10**^**–35**^
D × T	12	108	1.157	**5.16 × 10**^**–13**^	1.187	**8.79 × 10**^**–09**^
D × G	12	108	0.255	**0.015**	0.577	**8.26 × 10**^**–05**^
D × G × T	24	108	0.266	**0.002**	1.005	**2.23 × 10**^**–10**^
Error for between subject terms (d.f. = 18)			0.387		0.259	
Error for within subject terms (d.f. = 108)			0.115		0.103	

The table reports mean squares for each effect with the *P*-value of the associated F-test and degrees of freedom (d.f.) for the numerator (d.f.n) and denominator (d.f.d).

Significant tests are in bold.

The pH of the testa increases throughout time in all treatments, as shown in the significance of the Day factor and Day by the Treatment factor in Table 1. The higher increase concerning the value of day zero occurred at the end of the experiment, being on average 87.38, 80.92, and 76.12% for T1, T2, and T3, respectively (Fig. 1, Fig. S1). Higher order interactions for this trait are all significant: Day by Genotype, Day by Treatment by Genotype, respectively, reflecting genetic variation for the increase of testa pH through fermentation in each treatment (Table 1, Table S2). Thus, an increase in the average temperature of T3 was related to higher decreases in nib pH and lower increases in testa pH throughout fermentation (Fig. 1).

### Cd concentration of cacao beans in response to temperature-controlled fermentation

**Figure 2:**
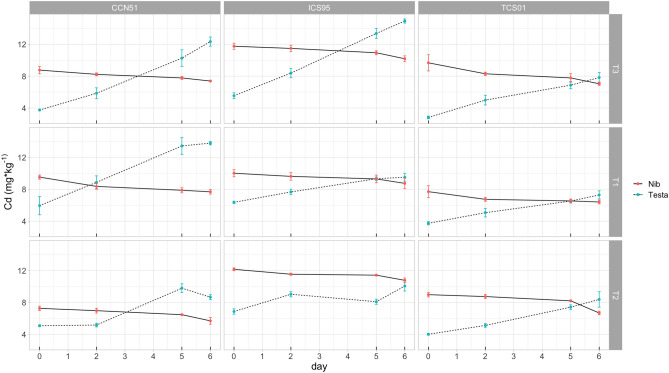
Time course for the Cd concentration (estimated on days 0, 2, and 6) of cacao bean tissues (nib and testa) of three genotypes (CCN 51, ICS 95, TCS 01) fermented under three temperature profiles (T) for 6 days. Means (dots) for three sample units and standard errors are plotted.

The RMANOVA for the nib Cd concentration showed a significant reduction throughout fermentation (significance for the Day factor, Table 2, Fig. 2, Fig. S2). Notwithstanding, none of the higher-order interactions with the Day were significant. Treatment, Genotype, and the Treatment by Genotype interaction effect significantly differed in the mean nib Cd concentration. The last might reflect variation in the initial Cd concentrations of the unfermented beans (Table S2). The primary source of this variation was the Genotype effect, whose means square variance was higher than the other sources of variance, followed by the Day and Treatment effect (Table 2).

**Table 2:** Repeated-measures ANOVA for the Cd concentration (estimated on days 0, 2, 5, and 6; see “Methods” section) of cacao bean tissues of three genotypes (G) fermented under three temperature profiles (T) for 6 days (D).

Effect	d.f.n.	d.f.d.	Nib Cd	*P*	Testa Cd	*P*	ITF	*P*
T	2	18	7.259	**0.007**	7.600	**0.015**	0.150	**0.010**
G	2	18	105.152	**2.60 × 10**^**–10**^	110.156	**1.48 × 10**^**–9**^	1.306	**3.37 × 10**^**–8**^
G × T	4	18	12.299	**9.83 × 10**^**–5**^	24.522	**5.81 × 10**^**–6**^	0.137	**0.005**
D	3	54	13.609	**1.76 × 10**^**–11**^	167.599	**9.35 × 10**^**–32**^	3.666	**1.07 × 10**^**–32**^
D × T	6	54	0.321	0.187	7.517	**3.68 × 10**^**–8**^	0.068	**3.39 × 10**^**–4**^
D × G	6	54	0.224	0.338	5.676	**1.51 × 10**^**–6**^	0.182	**2.18 × 10**^**–9**^
D × G × T	12	54	0.244	0.291	3.907	**2.08 × 10**^**–6**^	0.042	**0.002**
Error for between subject terms (d.f. = 18)			1.101		1.427		0.025	
Error for within subject terms (d.f. = 54)			0.190		0.663		0.013	

The table reports mean squares for each effect with the *P*-value of the associated F-test and degrees of freedom (d.f.) for the numerator (d.f.n) and denominator (d.f.d).

Significant tests are in bold.

**Figure 3:**
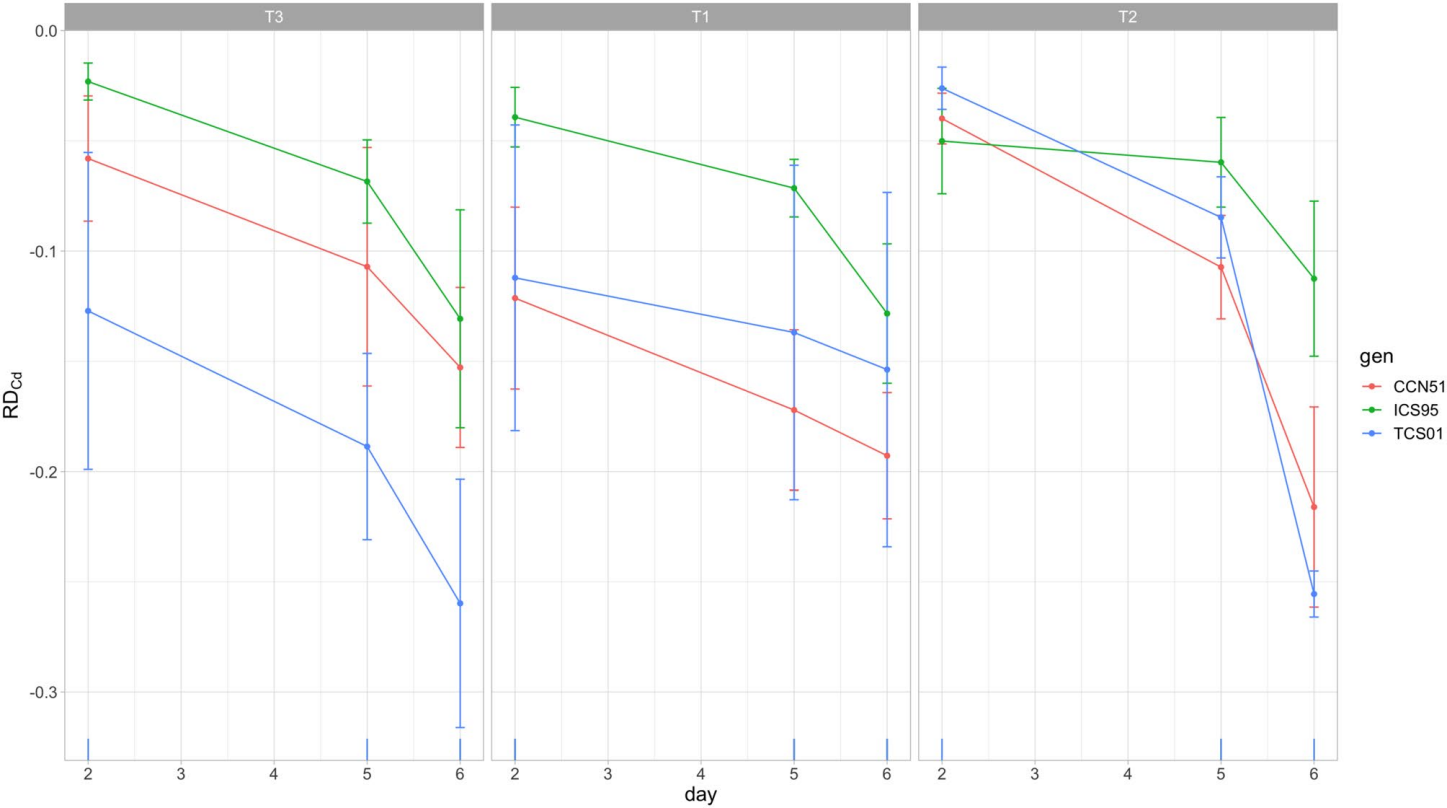
Time course for the relative decrease of Cd concentration (RD_Cd_, estimated on days 2, 5, and 6) of the cacao nib of three genotypes (CCN 51, ICS 95, TCS 01) fermented under three temperature profiles (T) for 6 days. Means (dots) for three sample units and standard errors are plotted.

Thus, the RMANOVA for the relative decrease of nib Cd concentration throughout fermentation (RD_Cd_, Table 3) showed a statistical significance of the Day factor and all its higher order interactions except Day by Genotype interaction (Table 3). In particular, the significant interaction Day by Genotype by Treatment showed genetic variation for the relative decrease of Cd concentration throughout fermentation (Table 3, Fig. 3, Fig. S3). Thus, on Day 6 of fermentation, genotypes had a statistically significant reduction for T1, T2, and T3 of 19.28, 21.61 and 15.28% (CCN51); 12.83, 11.25 and 13.07% (ICS95), and 15.37, 25.55 and 25.98% (TCS01), respectively (Table S2). An a posteriori multiple contrasts showed that TCS 01 and CCN 51 have no statistically significant differences in their mean relative decrease of 22.30 and 18.70%, respectively (Tukey HSD, *P* = 0.6081), followed by ICS 95 (12.4%, Tukey HSD, *P* < 0.05). These results showed that genetic variation played an essential role in the reduction of Cd through fermentation, and that cannot be expected as a universal reduction factor for the mitigation of Cd for all genotypes.

**Table 3:** Repeated-measures ANOVA for the relative changes in Cd concentration (estimated on days 2, 5, and 6; see “Methods” section) of cacao bean tissues of three genotypes (G) fermented under three temperature profiles (T) for 6 days (D).

Effect	d.f.n	d.f.d	RD_Cd_	*P*	RI_Cd_	*P*
T	2	18	0.0032	0.789	4.922	**2.87 × 10**^**–5**^
G	2	18	0.0390	0.080	1.364	**0.014**
G × T	4	18	0.0085	0.645	0.343	0.281
D	2	36	0.0854	**8.38 × 10**^**–15**^	4.987	**5.14 × 10**^**–14**^
D × T	4	36	0.0055	**0.001**	0.42	**3.56 × 10**^**–4**^
D × G	4	36	0.0016	0.179	0.278	**0.005**
D × G × T	8	36	0.0026	**0.017**	0.083	0.257
Error for between subject terms (d.f. = 18)			0.0134		0.249	
Error for within subject terms (d.f. = 36)			0.0009		0.062	

The table reports mean squares for each effect with the *P*-value of the associated F-test and degrees of freedom (d.f.) for the numerator (d.f.n) and denominator (d.f.d).

Significant tests are in bold.

**Figure 4:**
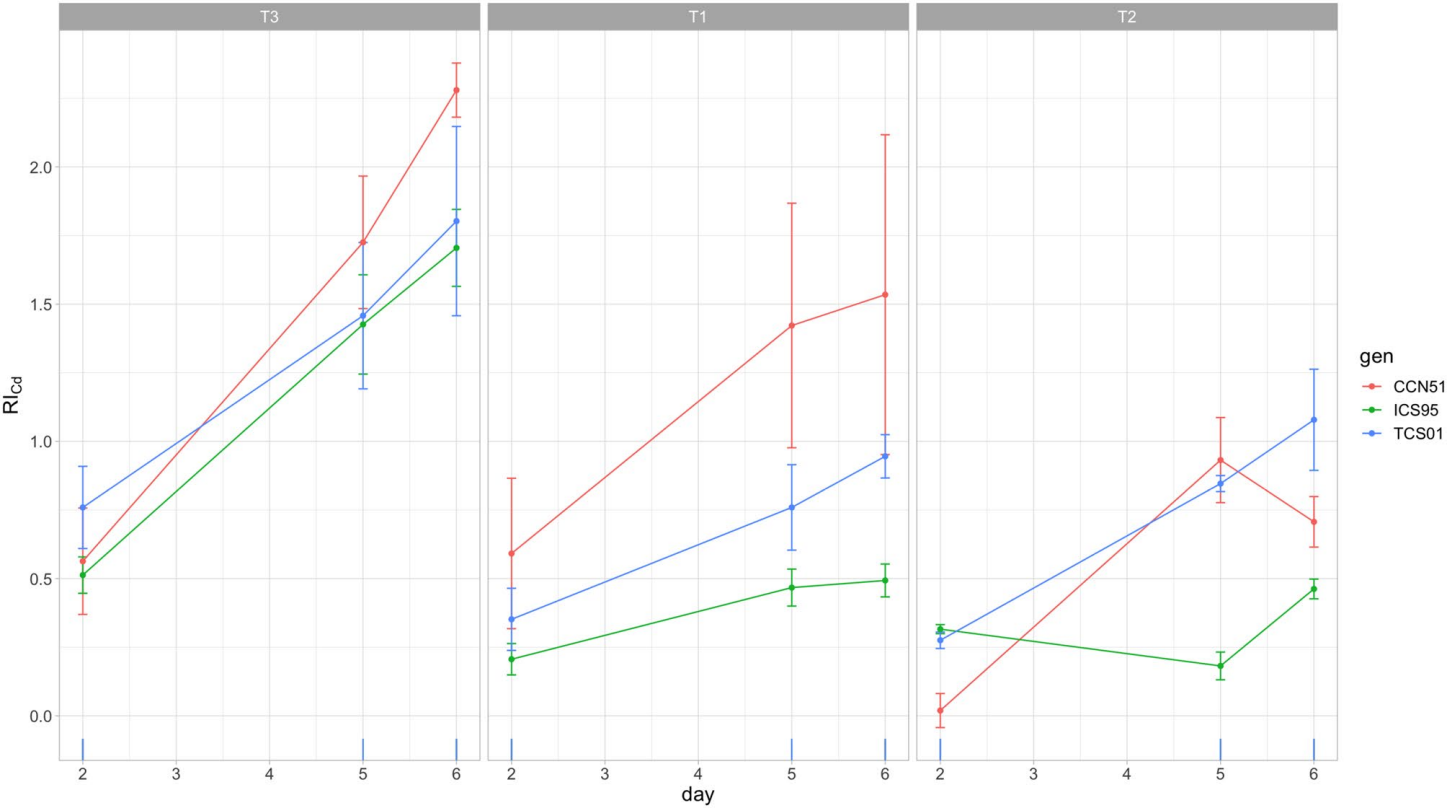
Time course for the relative increase of Cd concentration (RI_Cd_, estimated on days 2, 5, and 6) of the cacao testa of three genotypes (CCN 51, ICS 95, TCS 01) fermented under three temperature profiles (T) for 6 days. Means (dots) for three sample units and standard errors are plotted.

Results of the RMANOVA for the testa Cd concentration showed a statistically significant increase throughout fermentation (Table 2, Fig. 2, Fig. S2). The higher-order interactions with Day were significant, reflecting genetic variation for the increase of testa Cd concentration throughout fermentation in each treatment; this result was almost similar to analyzing the relative increase of testa Cd (RI_Cd_), which showed statistically significant differences for all higher interactions but Day by Genotype by Treatment (Table 3, Fig. 4). Thus, at Day 6 of fermentation, genotypes had a statistically significant increase of the testa Cd concentration for the T1, T2 and T3 of 153.50, 70.70 and 228.00% (CCN51); 49.30, 46.20 and 170.50% (ICS95), and 94.50, 107.90 and 180.20% (TCS01), respectively (Table S2, Fig. 4, Fig. S4). An a posteriori multiple contrasts showed that CCN51 had the higher mean (150.70%) significant increase (Tukey HSD, *P* < 0.05) followed by TCS 01 (127.60%) and ICS 95 (88.70%).

### Cd translocation from nibs to testa

**Figure 5:**
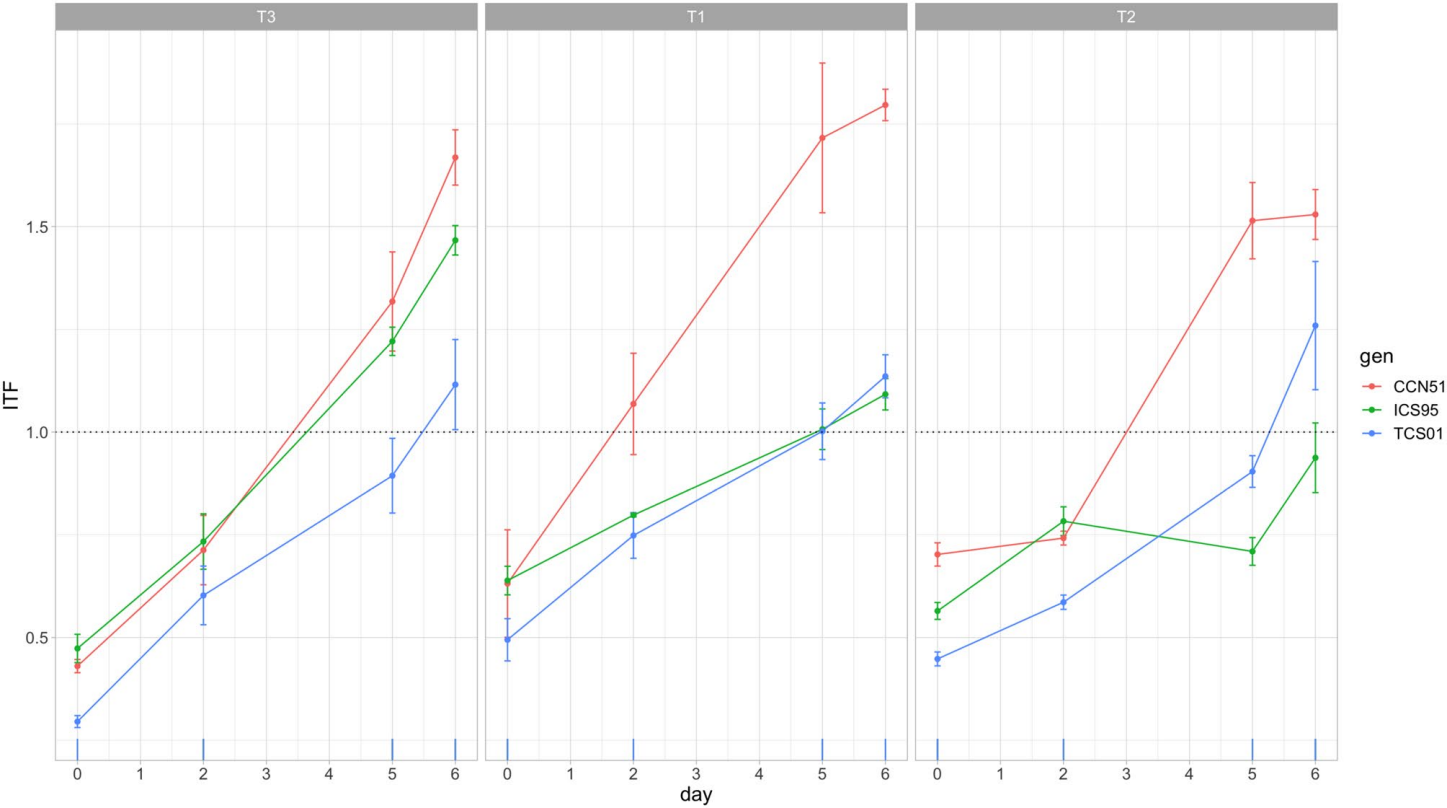
Time course for the internal translocation factor (ITF) of cacao bean tissues of three genotypes (CCN 51, ICS 95, TCS 01) fermented under three temperature profiles (T) for 6 days. Means (dots) for three sample units and standard errors are plotted. Reference horizontal dotted line indicates equal testa and nib Cd concentrations; higher values than 1 indicate a higher testa than nib Cd concentration.

The RMANOVA for the internal translocation factor (ITF) throughout fermentation showed a statistical significance of the Day factor and all its higher-order interactions (Table 2). In particular, the significant interaction Day by Genotype by Treatment showed genetic variation for the translocation of Cd from nibs to testa (Table 2). The ITF increased monotonically in almost all treatments (Fig. 5, Fig. S5). Figure 5 shows that in early-stage fermentation (before day 4), the increase of the ITF was below 1.0 for almost all genotypes in all treatments, which means Cd concentration for testa were bellow of the nibs. Values of ITF were higher than 1.0 for later-stage fermentation, indicating higher testa Cd concentrations compared to the nibs. Thus, at Day 6 of fermentation, genotypes had a statistically significant mean increase of ITF (Tukey HSD, *P* < 0.001) for the T1, T2, and T3 of 1.796, 1.529, and 1.668 (CCN51); 1.092, 0.937 and 1.467 (ICS95), and 1.136, 1.259 and 1.115 (TCS01), respectively. An a posteriori multiple contrasts showed that CCN 51 has the highest mean ITF (1.341, Tukey HSD, *P* < 0.0001), followed by ICS 95 (0.972) and TCS 01 (0.916) (Tukey HSD, *P* = 0.442).

All genotypes in all treatments had a significant decrease in nib Cd concentration between days 0 and 6 (Tukey HSD, *P* < 0.01; Table S2), which means extensive fermentation beyond the fifth day favors a decrease of nib Cd concentration. Notwithstanding, the analysis of mean Cd concentrations for each treatment showed that only T3 (Tukey HSD, *P* < 0.05) and T2 (Tukey HSD, *P* < 0.001) are temperature profiles able to further reduce Cd concentration significantly from day 5 to 6. The testa Cd concentration increased statistically significantly between days 0 and 6 for all genotypes in all treatments (Tukey HSD, *P* < 0.001; Table S2), meaning extensive fermentation beyond the fifth day favors an increase of testa Cd concentration. However, the analysis of mean Cd concentrations for each treatment showed that only T3 (Tukey HSD, *P* < 0.01) is the temperature profile able to further increase testa Cd concentration significantly from day 5 to 6.

### Correlation between nib pH and Cd concentration of cacao beans

A positive significant correlation was observed only for the nib pH and nib Cd concentration in T2 (Pearson *r* = 0.54 [0.26, 0.74, 95% CI]; *P* = 0.009) and for nib pH and RD_Cd_ in T3 (Spearman *rho* = 0.77 [0.57, 0.88, 95% CI]; *P* < 0.01). The ITF was negatively correlated with the nib pH only for T3 (Spearman *rho* =  − 0.58 [− 0.77, − 0.30, 95% CI]; *P* = 0.004).

### Sensorial analysis

A Non-Metric Multidimensional Scaling^32^ visualized the differences between genotypes for the flavor profile in each temperature treatment. These differences between genotypes were subsequently evaluated with an analysis of similarities (ANOSIM^33^). A multivariate analysis of indicator attributes (flavors) was carried out in each temperature treatment to determine those that occur most frequently^34^.

**Figure 6:**
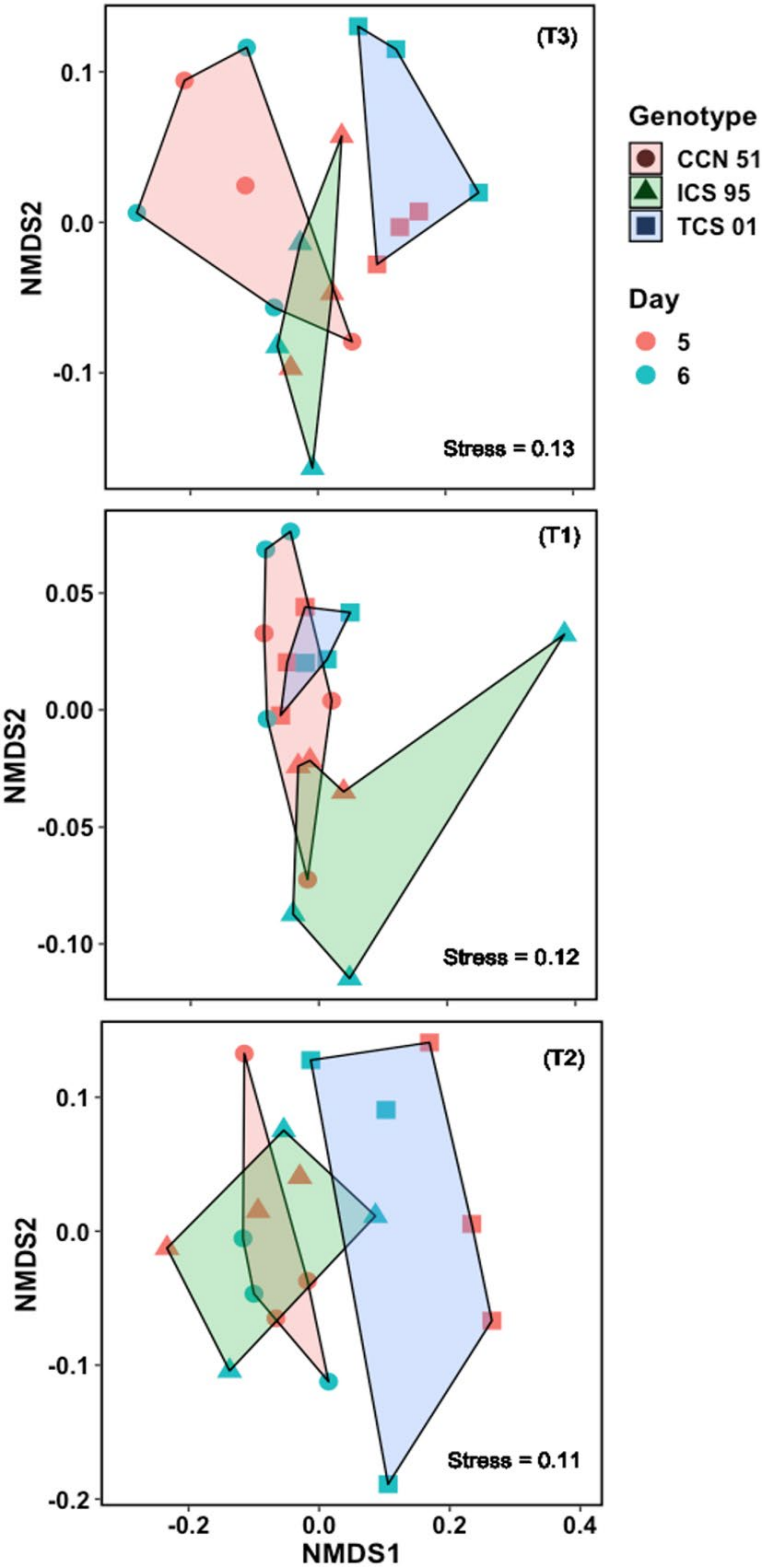
A non-metric multidimensional scaling (NMDS) plot for T3, T1, and T2. The NMDS was constructed with weighted Bray–Curtis metrics based on the intensity of flavor attributes. The stress value for all treatments was lower than 0.13, which showed that a higher-dimensional solution is unlikely to add to the overall picture^33^. Only clustering by genotypes (T3: R = 0.52, *P* = 0.0001; T1: R = 0.13, P = 0.0427; T2: R = 0.30, *P* = 0.0024) and not the days of fermentation was supported by ANOSIM.

**Figure 7:**
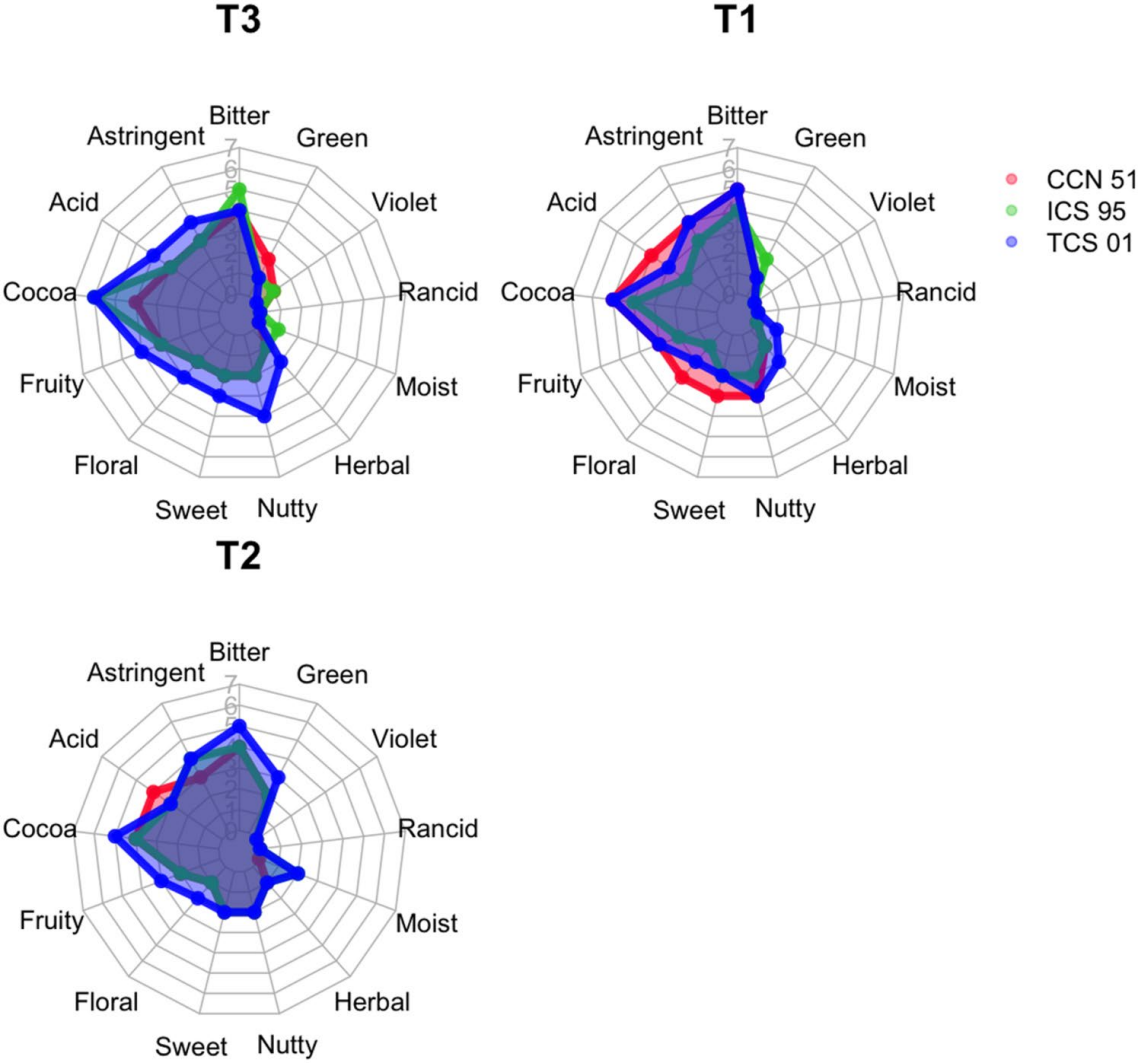
Sensory profiles (for day 6 of fermentation) with the scores for the intensity of flavor attributes of three cacao genotypes (CCN 51, ICS 95, TCS 01) fermented under three temperature profiles (T3, T2, T1).

The ANOSIM analysis for T1 showed significant differences (R = 0.13, *P* = 0.0427) for the sensory profiles of the genotypes (Figs. 6, 7), while there was no significant difference for the days of fermentation. The TCS 01 and CCN 51 genotypes overlap in the flavor profiles, while the ICS 95 genotype presents a more differentiated profile from the other genotypes (Figs. 6, 7). For T2, there were significant differences in the sensory profile of the genotypes (R = 0.30; *P* = 0.0024) but not for the day of fermentation (Figs. 6, 7). The TCS 01 genotype presents a more significant difference in the sensory profile concerning the similarity of the profile in the CCN 51 and ICS 95 genotypes. Figure 6 shows the separation of the sensory profiles for T3. There were significant differences between the flavor profiles (R = 0.52, *P* = 0.0001) but not for the days of fermentation. The TCS 01 genotype shows differences in the flavor profile concerning the ICS 95 and CCN 51 genotypes, while these two slightly overlap flavor profiles (Figs. 6, 7).

The indicator flavor test showed that in T1, the CCN 51 genotype is the only one that presented a significant difference (*P* = 0.0362) in the frequency of occurrence of acid taste. For T2, the TCS 01 genotype was the only one that presented a significant difference in the frequency of appearance of fruity (*P* = 0.0028), floral (*P* = 0.0094), and nutty (*P* = 0.0355) flavors. In T3, the TCS 01 genotype presented a significant difference in the frequency of appearance of the fruity (*P* = 0.0138), nutty (*P* = 0.0013), floral (*P* = 0.0099), sweet (*P* = 0.0074), and cocoa (*P* = 0.0249) attributes. The ICS 95 genotype presents a significant difference in bitter taste (*P* = 0.035), and for CCN 51, no attributes with a significant difference were found.

## Discussion

This study underscores the current challenges in cacao fermentation, particularly the critical role of temperature. The current investigation showed that temperature is instrumental in lowering the nib’s pH values, demonstrating a direct association between these variables that favors Cd migration from nibs to testa without compromising flavor quality. In the following, we will discuss our results in terms of changes in temperature and their effects on the beans’ pH, Cd, and flavor quality.

### Temperature and variation of nib and testa pH

Our research findings indicate a decrease in nib pH and an increase in testa pH during fermentation, consistent with previous studies^15,20,23,35,36^. The decline in nib pH might be due to the rise in acetic acid after the second day of fermentation, leading to its diffusion from pulp to nib^23,29^ in all cacao genotypes. A more pronounced decrease in pH was observed with prolonged fermentation and higher temperatures, similar to other studies on spontaneous cacao fermentation or fermentation-like incubation^15,18^. However, lower activity of lactic and acetic acid bacteria due to limited substrate availability might limit the migration of metabolites to the seeds, causing an increase in pH in the final stages of prolonged fermentation^35,37^ in our study that were more pronounced in low mean temperature (T1).

Furthermore, sustaining a controlled temperature from the fourth day of fermentation in the 45–48 °C range and not allowing the temperature to be maintained at 48 °C for a long time could favor a lower mean pH value (4.66 in T3). Interestingly, the current study’s use of thermostatically controlled temperature probably favored the development of adequate concentrations of acidic-acetic bacteria, which prevented lowering the pH to threshold values that would negatively impact the flavor quality^23^. The current investigation did not measure bacterial populations and their metabolic products; further research is needed.

### Temperature, Cd of cacao beans, and fermentation time

Fermentation of cacao beans at high temperatures, as those experienced in T2 and T3, experienced the maximum reduction of nib Cd concentrations using absolute and relative values (RD_cd_, Table S2), which represented together approximately a 19% increase in the ability to reduce nib Cd concentrations concerning T1. Thus, nib Cd concentration was correlated with nib pH in T2 and T3. Notwithstanding, the absence of a correlation between the nib pH and nib Cd concentration in T1, despite a significant reduction of Cd throughout fermentation, could be explained by the lack of a continuous decrease of pH with the fermentation mentioned above (see pH discussion).

Cacao genotypes varied in the reduction of Cd through fermentation, and a universal reduction factor for the mitigation of Cd for all genotypes was not observed. Other studies using the National cacao variety of Ecuador reported a moderate Cd reduction factor between 1.25 and 1.3 (20 and 23% of nib Cd reduction, respectively) using spontaneous fermentation^15,16^ or between 1.3 and 1.6 (23 and 37.5% of nib Cd reduction) using incubation like fermentation plus organic acid treatments, which reached higher temperatures between 45 and 65 °C, respectively (30,33), this higher Cd reduction associated with higher temperatures might compromise the flavor quality^22^. The current investigation showed that the TCS01 genotype reached the highest mean nib Cd reductions throughout fermentation, up to 27.09% (a 1.37 reduction factor) in T3. This reduction factor did not compromise the liquor’s flavor (see below).

In general, extensive fermentation beyond the fifth day favored a decrease in the nib Cd and an increase in testa Cd, which contrasts with lesser fermentation times (4–5 days) proposed in studies of the enhancement of fine flavor cocoa attributes under acidic reagents or spontaneous fermentations^26,27^. Our study showed that product safety requires fermentation until day 6 to reach low pH values and increase the mitigation of nib Cd. However, this extensive fermentation increased flavor quality (see below), representing an aggregate value of temperature-controlled fermentation.

Results for the Cd concentration of the testa revealed that rising the mean temperature throughout fermentation, as in T3, increased the ability of the testa to adsorb Cd; that is, the RI_cd_ increased by approximately 95% concerning T1 (Table S2). This increase in the testa Cd presented genetic variation throughout fermentation in each treatment (Table 2, Fig. 2, Fig. S2). The results of the translocation of Cd from nibs to testa confirmed this, and the same pattern of genetic variation was observed throughout fermentation in each treatment (Table 2). These results imply a genetic differential ability of the testa to function as a Cd adsorbent, which had been shown in another study on the role of cacao testa for heavy metal removal of acid solutions from industrial sources^38^.

These results emphasize that genetic variation for the adsorbent ability of testa played a role in the reduction of Cd through fermentation. However, results of the ITF showed that the genotype with the highest nib Cd reduction (TCS 01) was the lowest in transferring Cd to the testa (an approx. 32% reduction throughout treatments concerning the highest genotype, Fig. 5), possibly Cd leached to the mucilage (sweatings not measured in this study), as has been confirmed in other studies^15–17^.

Another study using incubation-like fermentation suggested that the leaching of Cd and other elements is an indication of partial retention of elements in the testa and that its sorption capacity might not have played a significant role in migrating the nib Cd^17^. However, using the same experimental approach had shown that the structural damage to the testa occurs after the first 48 h of fermentation and that before this period, the seed represents a closed system with no transport of matter between the incubation-medium outside and the nibs inside^39^. Then, in this period, the testa influences the course of the incubations through its barrier function. Our study evidence that the transfer of Cd between nibs and testa could occur in the first 2 days down the concentration gradient (see below) between these tissues (higher in nibs during the first 48 h in all treatments), being one of the driving forces behind the migration of Cd to the testa (Fig. 5, Fig. S5). It is clear from these data that the adsorbent ability of the testa played a role in increasing the transfer of Cd even when the concentration gradient changed (ITF > 1.0), and the Cd of the testa was higher than the nibs but continued to increase its Cd content until the final of fermentation in some genotypes (Fig. 5, Fig. S5).

The Cd adsorption capacity of the testa has been identified in other studies^15–18^. Cd speciation studies have shown that chelation with S-ligands as detoxification mechanisms is more activated in the nibs than in the testa^18^, which opened the reasonable speculation that other mechanisms binding the Cd with the cell wall are possibly acting in the testa^18^ as has been showed in different tissues of Cd hyperaccumulator plant species^40,41^. The development of breeding programs that are not mainly focused on improving characteristics for yield but for food safety (and sustainability) by taking advantage of genetic variation in the testa’s adsorbent ability can help meet market expectations.

### The Cd concentration gradient switched between the early and late stages of fermentation

This study evidenced that the migration of Cd from nib to testa is facilitated at the initial stage of fermentation by the concentration gradient (ITF < 1.0; Fig. 5), which means that the testa Cd concentration in all unfermented bean samples was lower than the nib Cd concentration (Fig. S2). This Cd migration down the concentration gradient early in fermentation could facilitate the reduction of nib Cd concentration in all treatments. However, at the final stages, a counter gradient variation of Cd was observed (ITF > 1.0; Fig. 5) in almost all genotypes in all treatments, but it was more significant with higher mean temperatures and lower mean pH values throughout fermentation (T3, Table S2).

A counter gradient variation throughout all fermentation has been reported and discussed by Vanderschueren et al.^15^ based on the findings in unfermented cacao beans of higher testa than nib Cd concentrations by other studies^42–44^. However, in our study, beans were peeled manually to separate testa from nib before oven-dried at 70 °C (see “Methods” section) for posterior Cd estimation, ensuring that Cd could not migrate between tissues in the process of drying, a possibility that would establish a reasonable doubt of a counter gradient variation of Cd in early stages of fermentation. Two lines of evidence are against a counter gradient variation in early-stage fermentation; first, another study consistently found a higher Cd concentration in the nibs than in the testa, using tissue separation before oven drying^45^. Second, higher fermentation temperatures like those experienced in the drying process had been shown to increase the mobilization of Cd^17,18^. More research using the standardization regarding this sample processing is in high demand to establish if not separating the nibs and testa before oven drying for chemical analysis is the factor behind the observation of a Cd mobilization against the concentration gradient in early-stage fermentation.

Vanderschueren et al.^15^ emphasize the need to decrease the pH in the nib to < 5.0 to generate the nib Cd migration to testa. The acidification-driven Cd migration hypothesis was tested in another study, showing that sufficient heat is necessary to mobilize Cd in cacao nibs during fermentation^16^. However, it has been pointed out that nib Cd mobilization during incubation-like fermentation may be related to other processes involving metabolites highly dependent on temperature, such as the phytate breakdown and release of phytate-bound Cd, with lower pH values after incubation^17,18^. More research using a high level of temporal resolution measurements of Cd throughout fermentation and more temperature profiles are needed due to the extent of the genetic variation of cacao to disentangle if there is a specific temperature threshold for the migration of nib Cd to the testa.

### Higher temperature and low nib pH increased the liquors’s flavor quality

The sensorial analysis showed that higher mean temperatures, such as those experienced in T3, could favor the development of high-quality flavors in the TCS 01 genotype. The T3 was designed with the aim that after the death of the embryo (in the first 2 days^21^), the temperature would not increase higher than the range of 45–48 °C, which is not favorable for the production of acetic acid due to the death of acetic bacteria^22,46^, the acid that is highly responsible for flavor formation^35^. Spontaneous fermentation studies have shown that a high-temperature range (45–50 °C) releases a lower heat rate to the medium, maintaining a maximum variation of 5 °C^25,47^. This isothermal temperature range^25^ would promote the degradation of flavonoids, reducing bitterness and astringency^48^ and promoting the development of fine-flavor cocoa sensorial notes^25,49^.

Other studies have reported that pH values between ca. 4.75 and 5.2 and temperatures below 40 °C indicate an excellent fermentation process in spontaneous fermentation^23,24,29^. This study demonstrated that sustaining a controlled temperature from the fourth day of fermentation in the range of 45–48 °C and not allowing the temperature to be maintained at 48 °C for a long time could favor a lower mean pH value (4.66) and significant reductions in the nib Cd throughout fermentation without affecting the adequate flavor formation as designed in T3.

### Controlled temperatures and innovation technologies

Inadequate control of temperature can lead to problematic fermentations, impacting microorganism development, fermentation kinetics, and, consequently, the final product flavor^20,22^. However, the potential of using thermostatically controlled temperatures in fermentation to inspire the development of energy-efficient innovations is not just promising but exciting. These innovations could potentially revolutionize the cacao industry, transforming cacao into a safe and high-flavor quality product, as demonstrated by fermenters with innovative temperature control systems using water flows that increase energy efficiency, developed in other food industries^50^.

## Conclusion

The current study evidenced that increasing the mean temperature throughout fermentation is instrumental in lowering the nib pH, demonstrating a direct association between these variables that favors Cd migration from nibs to testa (maximum mean RF = 1.37) and the organoleptic properties of the liquor. Sustaining a controlled temperature from the fourth to the sixth day of fermentation in the range of 45–48 °C and not allowing the temperature to be maintained at 48 °C for more than 24 h can increase the migration of nib Cd to the testa. The migration of Cd occurred down the concentration gradient in early-stage fermentation and switched to a counter-gradient variation in the late stages of fermentation, probably extended by the adsorbent capacities of the testa. Our study did not measure fermentation sweating before and after the first 48 h of fermentation (the time framework of the testa barrier function); studies in this direction increasing the measurements of Cd and pH throughout fermentation time would help to explain the role of the testa increasing the mitigation of nib Cd. However, to accurately determine concentration gradients, it is essential to separate bean tissues in nibs and testa before chemical analyses to avoid cadmium migration, which could be favored for higher temperature conditions in the drying process. A complex genotype by temperature treatment interaction was evident, and it cannot be expected to obtain a universal percentage of decrease of Cd or a specific sensory profile for all cacao genotypes with fermentation; more research is needed to obtain a temperature profile according to each genotype’s biochemical response and the internal Cd translocation factor. Selecting genotypes with highly Cd adsorbent testas is a strategy that, combined with controlled-temperature fermentation, can help achieve a product that complies with EU regulations. Thermostatically controlled temperatures can be a sustainable technology for designing fermentation systems in small and medium-scale cacao production farms, helping to assure product safety and quality.

## Methods

### Genotypes

Ripe cacao fruits from three high-yielding genotypes, TCS01, ICS95, and CCN51, representing cacao-experimental local production from the municipality of San Vicente de Chucurí, department of Santander, Colombia, were used. The samples were collected under the framework collection permit granted to AGROSAVIA under resolution No 1466 of 3 December 2014 of A.N.L.A. (Colombia). The genotypes differed in the average grain index: 2.6, 1.6, and 1.3 for TCS01, CCN51, and ICS95, respectively^51^. Grain size variation allowed us to observe how temperature affects the same mass in fermentation but with different surface-volume relationships (see treatments below). In general, bodies with a large surface area to volume ratio (i.e., small diameters) react at higher rates than monolithic materials since a larger surface is exposed to react^52^.

### Fermentation essays

**Figure 8:**
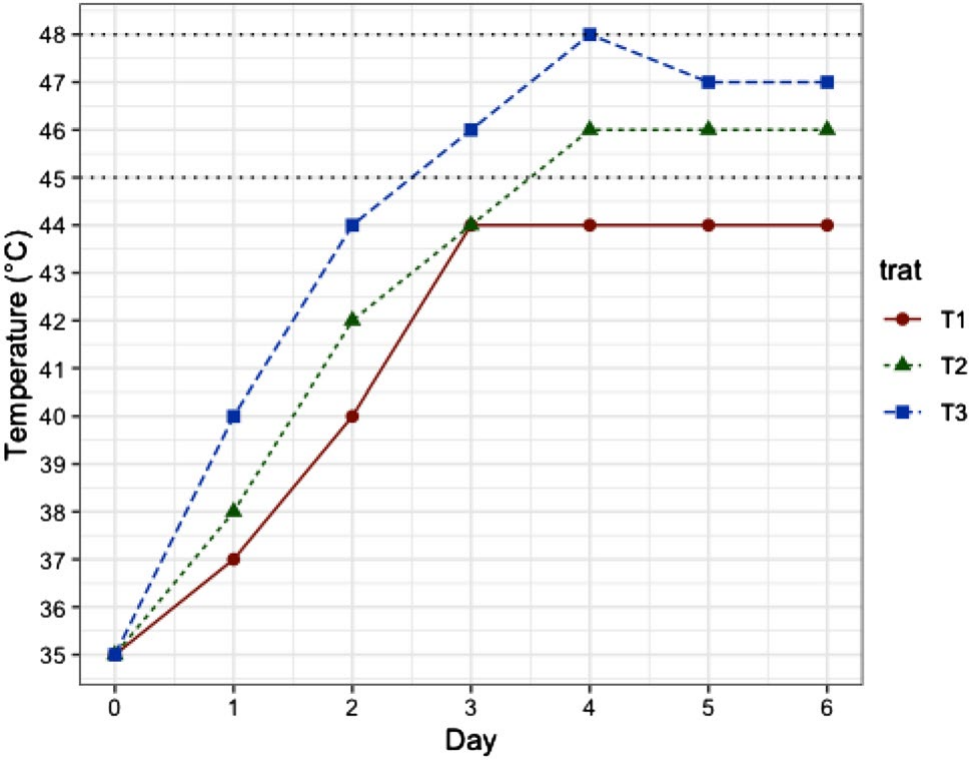
Temperature profiles (treatments) were used in the experiment. Treatments were designed to increase the average fermentation temperature, aiming to decrease the nib pH from 6.5–7 to 4.5–5.0 due to the dominant presence of acetic acid bacteria in the aerobic phase (after 48 h)^35,46^, but without increasing the temperature higher than the range of 45–48 °C (horizontal dotted reference lines), which are not favorable for the production of acetic acid, due to the death of acetic bacteria, an acid that is highly responsible for flavor formation.

**Figure 9:**
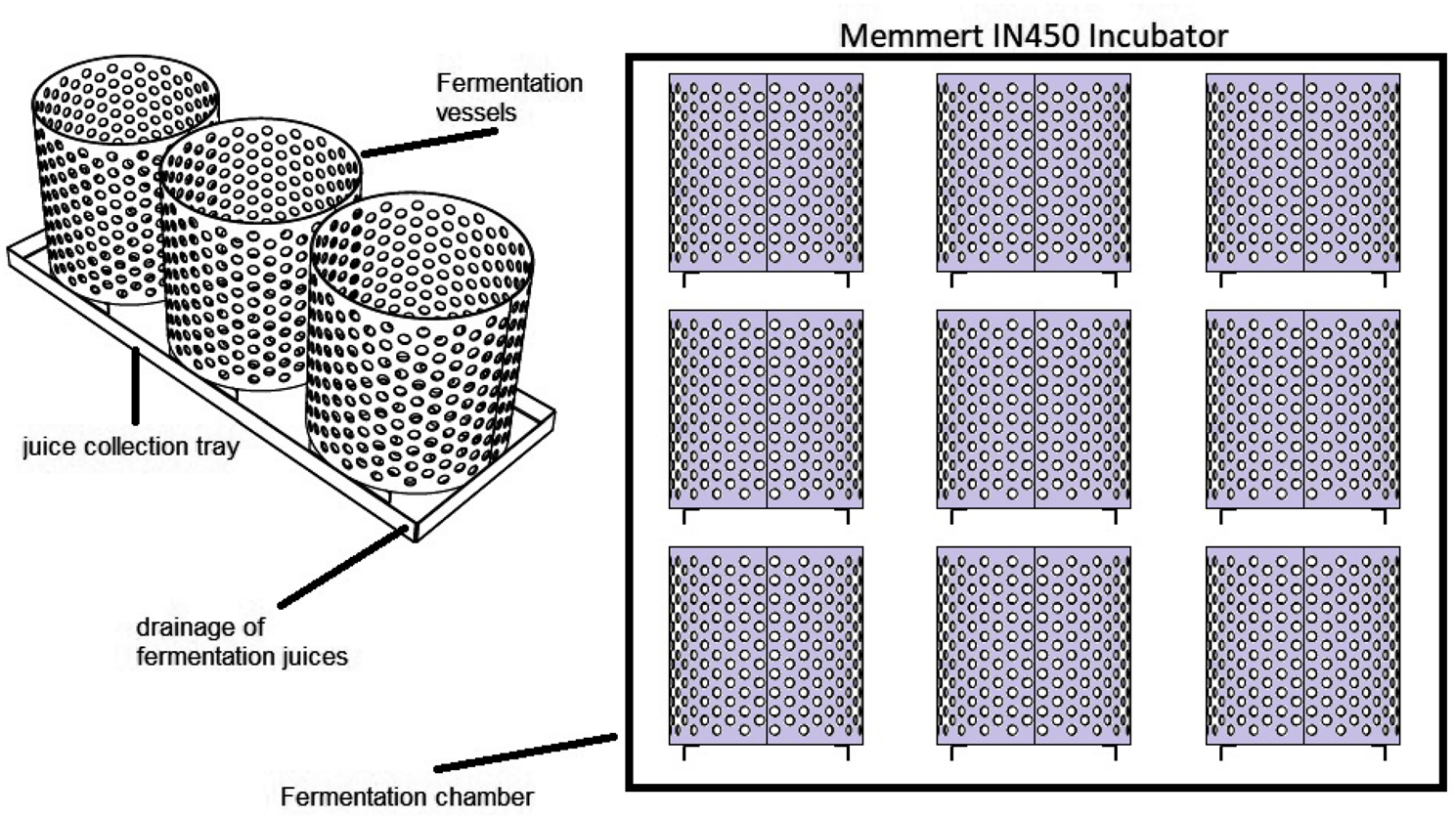
Depiction of fermentation stainless vessels, juice collection trays, and the experimental arrangement of the incubator. Experimental units consisted of batches of 3.8 kg of wet cacao beans. Six experimental units from each genotype were randomly assigned to two juice collection trays (three experimental units per tray). The sampling unit per genotype was a composite sample of two experimental units assigned randomly, one from each juice collection tray for a total of three sample units per genotype (see “Methods” section), depicted in the incubator.

Fermentation was carried out in the lab facilities of La Suiza Research Center (Agrosavia), Rionegro, Santander, Colombia (coordinates 7° 22ʹ 13ʹʹ, − 73° 10ʹ 39ʹʹ), using an incubator (Memmert IN450). The incubator used three different temperature profiles (i.e., treatments hereafter; Fig. 8 and Table 4; see the rationally behind treatments in Supplementary Information) aimed to achieve variation in the final pH that allows us to test its role in Cd migration from nib to testa, the increase in average fermentation temperature in spontaneously fermented cocoa beans is related to the decrease in nib pH from 6.5–7.0 to 4.5–5.0 due to the dominant presence of acetic acid bacteria in the aerobic phase (approx., hour 48 of the fermentation^35,46^). Fermentation essays were conducted to study the effect of the different temperature profiles on the distribution of Cd between the cacao’s testa and nib tissues and the sensory quality of fermented beans. The three treatments were applied one at a time to the three genotypes using the incubator for easy treatment application. Experimental units consisted of batches of 3.8 kg of wet cacao beans fermented in stainless vessels, with perforated holes throughout their surface to enhance the fermentation sweating drainage and the heat transfer to the fermentation mass (Fig. 9). Six experimental units from each genotype were used and assigned randomly to two juice collection trays (three experimental units per tray). The sampling unit per genotype was a composite sample of two experimental units assigned randomly, one from each juice collection tray, for a total of three sample units per genotype (14, 25 and 36 samples). Fermentation juice was drained through holes in the collection trays connected with an infusion hose to collection recipients inside the incubator (Fig. 9). Fermentation for each temperature profile was given 6 days (144 h) and with a frequency of turning every 24 h from the second day (48 h) until the fifth day (120 h) with a stainless-steel shovel for 30 s each time.

**Table 4:** Controlled temperature (T) profiles (°C) for 6 days (144 h) of fermentation.

Hour	T3	T1	T2
0	35	35	35
24	40	37	38
48	44	40	42
72	46	44	44
96	48	44	46
120	47	44	46
144	47	44	46

### Physicochemical variables

#### pH of cacao beans

Monitoring pH was measured by sampling 50 ± 0.01 g of cacao beans per sample from zero hours every 24 h until the end of fermentation (except for Cd concentration; see below). Cacao beans were manually separated in nib and testa, and the AOAC 970.21^53^ potentiometric method for the pH of cacao products was used to determine their pH using a Hanna (HI 5221, Woonsocket, Rhode Island, United States) pH meter.

#### Cd concentration of cacao beans

The Cd concentration was evaluated by sampling 100 ± 0.01 g of cacao beans per sample on day zero, two (48 h), five (120 h), and six (144 h). The mucilage in cacao beans was removed using paper towels, and the nib and testa tissues were manually separated. All cacao tissues were oven-dried to constant weight for at least 72 h at 70 ± 0.1 °C, and the Cd concentration of cacao beans (nib and testa) was analyzed in the analytical chemistry laboratory of Tibaitatá Research Center (Agrosavia), Mosquera, Cundinamarca, Colombia (coordinates 4° 41ʹ 45.2ʹʹ, 74° 12ʹ 19.7ʹʹ) following the methodology of Rodríguez-Giraldo et al.^54^. The laboratory has accreditation ONAC ISO/IEC 17025:2017^55^ for quantification of cadmium in cacao beans (AOAC 2013.06^56^).

#### Amount of reduction of Nib Cd in response to fermentation

The reduction of Cd in response to fermentation was estimated with the relative decrease (RD_Cd_) to compare the value of unfermented (*u*) and fermented nibs (*j*) for each sample (*i*) while considering the value in the unfermented state for the day 2, 5 and 6 of fermentation. For each genotype, the mean RD_Cd_ for a given day was:

1 $${\overline{RD}}_{Cd}=\sum_{i=0}^{n}(({x}_{ij}-{x}_{iu})/{x}_{iu}))/n.$$

#### Amount of increase of testa Cd in response to fermentation

The increase of Cd in response to fermentation was estimated with the relative increase (RI_Cd_) to compare the value of unfermented (*u*) and fermented nibs (*j*) for each sample (*i*) while considering the value in the unfermented state for the day 2, 5 and 6 of fermentation. For each genotype, the mean RI_Cd_ for a given day was:

2 $${\overline{RI}}_{Cd}=\sum_{i=0}^{n}(({x}_{ij}-{x}_{iu})/{x}_{iu}))/n.$$

#### Cd translocation from nib to testa

An internal translocation factor (ITF) due to fermentation was estimated as the Cd concentration ratio between testa (*t*) and nib (*N*) tissues for day 0, 2, 5, and 6 of fermentation. For each genotype, the mean ITF for a given day was:

3 $$\overline{ITF }=\sum_{i=0}^{n}{\left[\frac{{Cd}_{t}}{{Cd}_{N}}\right]}_{ij}/n.$$

The mean relative increase of the ITF for a given genotype and day was:

4 $${\overline{R}}_{ITF}=\sum_{i=0}^{n}{\left[\frac{{Cd}_{t}}{{Cd}_{N}}\right]}_{ij}-{\left[\frac{{Cd}_{t}}{{Cd}_{N}}\right]}_{iu}/n.$$

#### Sensory analysis

The sensorial traits were evaluated by sampling 150 beans per sample on fermentation’s fifth and sixth days. The analysis of sensorial traits considered the relevant guidelines and regulations for the sensory evaluation of foods and cocoa liquor carried out in the Laboratory of Analytical Chemistry of the La Suiza Research Center following the ICONTEC GTC 165 (2014) and the ICONTEC NTC 3929 (2009) norms. Sensory analysis was performed on 54 samples of cocoa liquor (three genotypes by three treatments by two evaluation times by three samples). First, the cocoa nibs were obtained after roasting the grain for 12 min at 110 ± 0.1 °C in a rotary roaster (KAFFEMAT, model 1220, Bogotá, Colombia), then the shelling process was carried out with the help of a crusher (COCOATOWN, CocoaT Cracker, United States) and a sheller (COCOATOWN, CocoaT Winnower, United States). Finally, the nibs were ground in a granite stone grinder (COCOATOWN, Melanger ECGC-12SLTA, United States) until liquor samples with particle sizes between 25 and 30 μm were obtained. The cocoa liquors were melted at 55 ± 0.1 °C and deposited in covered containers with a net content of 5 ± 0.01 g to be delivered (after being coded) to five trained evaluators (ages between 25 and 38 years) who are part of the sensory evaluating panel of cocoa liquor from the La Suiza Research Center, in 18 tasting sessions evaluating six liquors per session, the evaluation of each liquor was carried out in duplicate to arrive at the sensory profile, including the following sensory attributes: basic flavors (astringency, bitterness and acidity); fine flavors (cocoa, fruity, floral, nutty, sweet, and herbal) and acquired flavors (moisture, rancid, violet and green). A Quantitative Descriptive Analysis (QDA) was applied to evaluate the ranges of flavor attributes on a scale of 0 to 10, where 0 represents absence, and 10 describes a high flavor intensity. Thus, we managed to identify the state of fermentation of the samples and possible foreign flavors, which can be the product of inadequate processing and drying or a low percentage of fermentation.

### Statistical analysis

#### Variation of traits in response to fermentation

All variables except sensorial traits were evaluated using Repeated Measures ANOVA (RMANOVA), which investigated the effect of the following sources of variation: Genotype (genetic differences in trait values), Day (variation of trait values during fermentation time), Treatment (variation of trait values in response to temperature profile), Genotype by Day (Genetic variation of trait trajectory during fermentation time), Genotype by Day by Treatment (Genetic variation of the trait trajectory during fermentation time depending on temperature profile). All analyses were performed using the software R v. 4.1.0 (R Core Team, 2021). The statistical tests used a *P*-value of 0.05. The results of interest from this analysis are for the effects of Day, the within-subject effect describing variation within and among fermentation trajectories and using a repeated measurements analysis of variance permitted to correct F values to account for the reduced degrees of freedom due to the autocorrelation of values through time. A posteriori multiple comparison of means was performed using Tukey’s Honestly Significant Difference test (P-value ≤ 0.05).

#### Correlation between nib pH and Cd concentration of cacao beans

The correlation between nib pH and the decrease of nib Cd, RD_Cd_, RI_Cd_, and ITF during fermentation for each treatment was tested using Pearson’s or Spearman’s correlation test when needed (P value ≤ 0.05).

#### Sensorial traits

All analyses were performed using the software R v. 4.1.0^57^ (R Core Team, 2021). A Non-Metric Multidimensional Scaling (NMDS) was performed using the *vegan* package of R (version 2.6-4^58^) to address the differences in the sensory profiles of genotypes in each treatment. The Bray–Curtis distance was used based on the occurrence of flavor traits in each genotype. Then, an analysis of similarities (ANOSIM^33^) was performed using the vegan package of R, in which the intensity in the presence of the different flavors was the response variable, and the predictor variable was the genotype or the fermentation day (five and six), allowing us to corroborate statistically, additional to the graphical results of the NMDS if the sensory profile change with the genotype or the number of days used for fermentation. We also performed a post-hoc comparison test, when ANOSIM for genotypes was statistically significant, to show which genotypes differ in the sensory profile using the *pairwiseAdonis* package of R^59^.

The *indicspecies* package of R (version 1.7.14^60^) was used to test which flavors are statistically significant indicators of the sensory profiles of each genotype in each treatment. The best temperature profile was evaluated by its ability to separate the genotypes’ sensory profiles and express more significant flavors. The statistical tests used a *P*-value of 0.05.

### Supplementary Information

Supplementary Information.

## Data Availability

All data generated or analyzed during this study are included in this published article (and its Supplementary Information files).
