# Supplementary material for: High temperature and nib acidification during cacao-controlled fermentation improve cadmium transfer from nibs to testa and the liquor’s flavor
Source: Sci Rep. 2024 May 28;14:12254. doi: 10.1038/s41598-024-62609-8 (PMC11133384; doi:10.1038/s41598-024-62609-8)
Supplement: Supplementary file 1 — Supplementary Information. [file 41598_2024_62609_MOESM1_ESM.pdf]

SUPPLEMENTARY INFORMATION

High temperature and nib acidification during cacao-controlled fermentation improve cadmium transfer from nibs to testa and the liquor's flavor

Iván D. CAMARGO[1*], Lucero G. RODRIGUEZ-SILVA[1], René CARREÑO-OLEJUA[1], Andrea C. MONTENEGRO[2], Lucas F. QUINTANA-FUENTES[3]

[1] Corporación Colombiana de Investigación Agropecuaria – AGROSAVIA, La Suiza Research Center – Km 32 Route to sea, 687527 Santander, Colombia.

[2] Corporación Colombiana de Investigación Agropecuaria – AGROSAVIA, Tibaitatá Research Center – Km 14 Route Mosquera-Bogotá, 250047 Cundinamarca, Colombia.

[3] Universidad Nacional Abierta y a Distancia, Facultad de Ingeniería. Calle 14 Sur No. 14 – 23 Barrio Restrepo, 111511 Bogotá, Colombia.

[*]Corresponding author

✉ Ivan D. CAMARGO: idcamargo@agrosavia.co

Rationality behind temperature profiles (treatments)

Three temperature profiles were established to obtain variations in the fermentation pH (Table 1). Thus, a temperature gradient was used for the death of the embryo to achieve a differential decrease in the pH of the nib, which will simulate the most favorable conditions reported in the literature for spontaneous fermentations:

- Embryo death occurs in the first 48 hours between 36-44 °C[1].
- After the death of the embryo, temperatures reach 45-50 °C until the end of fermentation[2–4].
- There are two main fermentation temperature phases. The exothermic phase (from day 0 to 4) with a higher metabolic activity increased from approx. 27 to 49 °C, while the isothermic phase (from day four on) with a lower variation in temperature remains constant between 45 and 50 °C[5].
- After the death of the embryo, temperature increments higher than the range of 45-48 C are not favorable to produce acetic acid due to the death of acetic bacteria[2,3].
- Try reaching phases below 35 °C for long periods has not resulted or has resulted in grain germination[2,6].
- Depending on the observed flavor, Small-scale fermentation can achieve good fermentation with a maximum temperature of 46 °C and 38-46 °C after the thirtieth hour[7].

Table S1. Abbreviations used in this paper.

Abbreviation	Variable
CCN 51	Cacao genotype
ICS 95	Cacao genotype
TCS 01	Cacao genotype
Cd	Cadmium
T1	Temperature treatment 1
T2	Temperature treatment 2
T3	Temperature treatment 3
RMANOVA	Repeated Measures Analysis of Variance
RD_{Cd}	Relative decrease of nib Cd concentration
RI_{Cd}	Relative increase of testa Cd concentration
RF	Reduction Factor
ITF	Internal Translocation Factor

Table S2. Cacao bean physicochemical traits of three genotypes (CCN 51, ICS 95, TCS 01) before (unfermented, day 0) and at the final (fermented, day 6) of fermentation in three controlled temperature treatments (T1, T2, T3). The table reports the means of three samples and 95% confidence intervals (in parentheses).

**Treatment**	**T1**			**T2**			**T3**		
**FT(1)**	**41.14 ± 3.84 °C**			**42.43 ± 4.39 °C**			**43.86 ± 4.74 °C**		
**Genotype**	**CCN 51**	**ICS 95**	**TCS 01**	**CCN 51**	**ICS 95**	**TCS 01**	**CCN 51**	**ICS 95**	**TCS 01**
**Nib pH**									
Unfermented	5.41 (4.96-5.86)	5.04 (4.59-5.49)	5.54 (5.09-5.99)	5.33 (4.88-5.78)	6.12 (5.67-6.56)	5.80 (5.35-6.25)	5.60 (5.15-6.05)	5.60 (5.15-6.05)	5.69 (5.24-6.14)
Fermented	5.01 (4.56-5.46)	5.50 (5.05-5.95)	5.80 (5.35-6.25)	5.22 (4.77-5.67)	5.87 (5.42-6.32)	5.62 (5.17-6.07)	5.05 (4.60-5.50)	4.78 (4.33-5.22)	4.05 (3.60-4.50)
**Testa pH**									
Unfermented	2.96 (2.55-3.36)	2.85 (2.45-3.26)	3.47 (3.06-3.87)	2.40 (1.99-2.80)	3.43 (3.02-3.83)	3.31 (2.90-3.71)	3.19 (2.79-3.60)	3.23 (2.83-3.64)	3.62 (3.22-4.03)
Fermented	5.54 (5.13-5.94)	5.04 (4.63-5.44)	6.80 (6.40-7.21)	5.30 (4.89-5.70)	4.67 (4.26-5.07)	6.54 (6.13-6.94)	6.65 (6.25-7.06)	6.62 (6.21-7.02)	4.43 (4.02-4.83)
**Nib Cd**									
Unfermented	9.53 (8.78-10.29)	10.02 (9.26-10.77)	7.71 (6.95-8.46)	7.27 (6.51-8.02)	12.16 (11.41-12.92)	8.98 (8.23-9.74)	8.76 (8.01-9.56)	11.75 (10.99-12.51)	9.67 (8.92-10.43)
Fermented	7.69 (6.93-8.45)	8.75 (7.99-9.50)	6.41 (5.66-7.17)	5.70 (4.95-6.46)	10.78 (10.03-11.54)	6.69 (5.93-7.44)	7.39 (6.63-8.15)	10.18 (9.43-10.94)	7.05 (6.29-7.80)
% RD_{Cd} (-)(2)	19.30 (28.80-9.73)	12.8 (22.4-3.28)	15.4 (24.90-5.82)	21.60 (31.20-12.05)	11.3 (20.8-1.70)	25.6 (35.10-16.00)	15.30 (24.8-5.72)	13.10 (22.6-3.51)	26.00 (35.50-16.42)
**Testa Cd**									
Unfermented	5.96 (4.90-7.03)	6.37 (5.30-7.44)	3.75 (2.68-4.81)	5.09 (4.02-6.16)	6.87 (5.81-7.94)	4.02 (2.95-5.08)	3.76 (2.69-4.82)	5.55 (4.48-6.62)	2.83 (1.76-3.90)
Fermented	13.79 (12.72-14.86)	9.52 (8.45-10.58)	7.30 (6.23-8.36)	8.67 (7.60-9.74)	10.06 (8.99-11.13)	8.39 (7.32-9.45)	12.33 (11.27-13.40)	14.91 (13.85-13.98)	7.83 (6.76-8.89)
% RI_{Cd} (+)(2)	153.50 (101.94-205.00)	49.30 (2.19-100.80)	94.50 (43.03-146.1)	70.70 (19.16-122.20)	46.20 (5.28-97.80)	107.90 (56.34-159.40)	228.00 (176.46-279.5)	170.5 (118.98-222.00)	94.50 (43.03-146.10)
**ITF**									
Unfermented	0.63 (0.48-0.78)	0.64 (0.49-0.79)	0.49 (0.35-0.64)	0.70 (0.56-0.85)	0.57 (0.42-0.71)	0.45 (0.30-0.6)	0.43 (0.28-0.58)	0.47 (0.33-0.62)	0.30 (0.15-0.44)
Fermented	1.80 (1.65-1.94)	1.09 (0.95-1.24)	1.14 (0.99-1.28)	1.53 (1.38-1.68)	0.94 (0.79-1.09)	1.26 (1.11-1.41)	1.67 (1.52-1.82)	1.47 (1.32-1.61)	1.12 (0.97-1.26)
% R_{ITF} (+)(2)	218.70 (143.18-294.00)	71.3 (4.23-147.00)	135.90 (60.38-212.00)	117.8 (42.27-193.00)	65.5 (10.04-141.00)	180.00 (104.44-256.00)	289.50 (213.98-365.00)	214.40 (138.80-290.00)	277.10 (201.53-353.00)

(1) Fermentation temperature as the mean (± standard deviation) of controlled temperature across six fermentation days.

(2) Relative change of the trait for day 6 of fermentation expressed as a percentage of decrease (nib means, and 95% CIs reported are negative) or increase (testa and ITF means, and 95% CIs reported are positive) concerning the unfermented state (see $\overline{RD}_{Cd}$, $\overline{RI}_{Cd}$ and $\bar{R}_{ITF}$ in methods).

Figure S1. Time course for the pH of cacao bean tissues of three genotypes (CCN 51, ICS 95, TCS 01) fermented under three temperature profiles (T) for six days. Three sample units (14, 25, 36) per genotype are plotted.

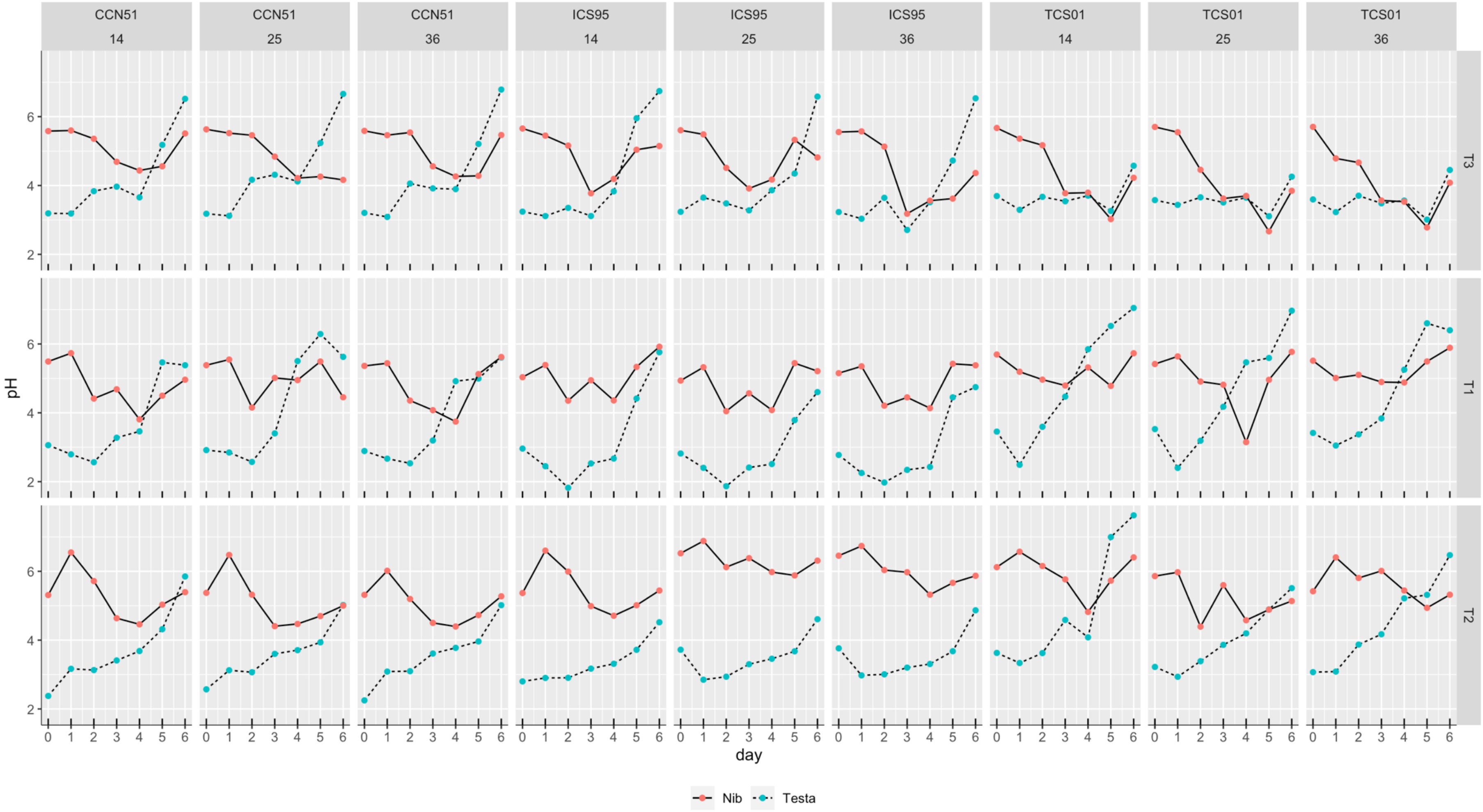

Figure S2. Time course for the Cd concentration (estimated on days 0, 2, and 6) of cacao bean tissues of three genotypes (CCN 51, ICS 95, TCS 01) fermented under three temperature profiles (T) for six days. Three sample units (14, 25, 36) per genotype are plotted.

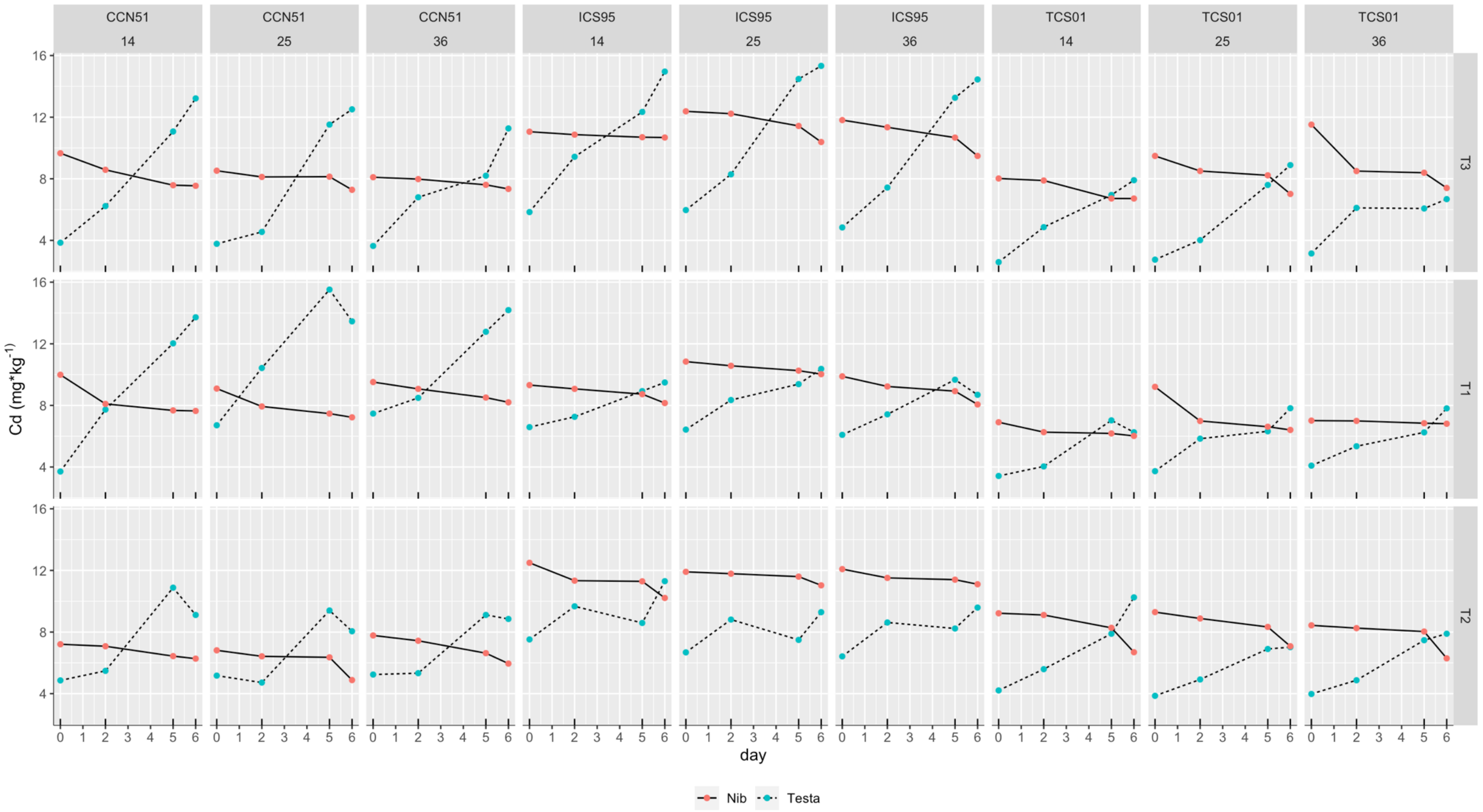

Figure S3. Time course for the relative decrease of Cd concentration (RD_{Cd}, estimated on days 2, 5, and 6) of the cacao nib of three genotypes (CCN 51, ICS 95, TCS 01) fermented under three temperature profiles (T) for six days. Three sample units (14, 25, 36) per genotype are plotted.

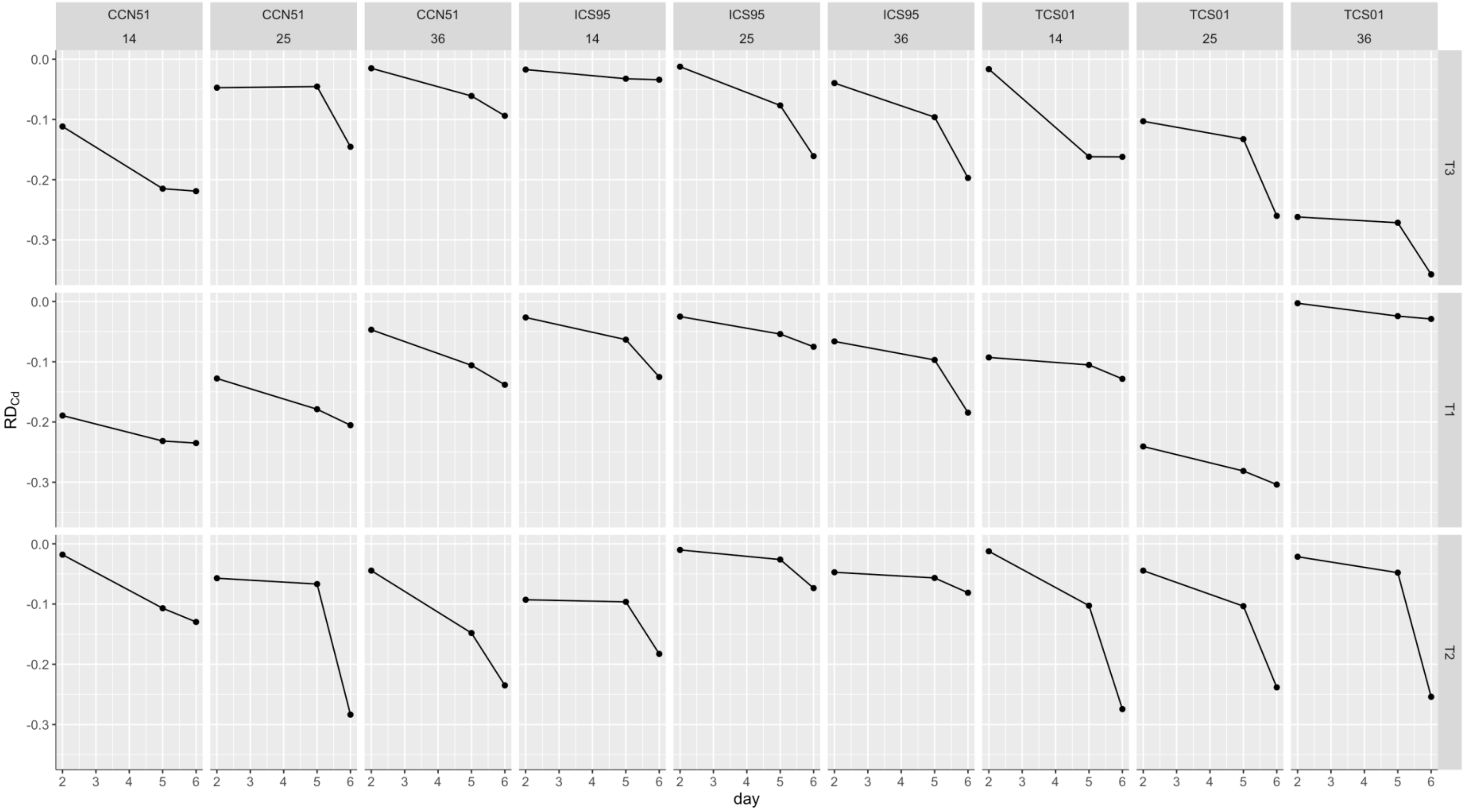

Figure S4. Time course for the relative increase of Cd concentration (RI_{Cd}, estimated on days 2, 5, and 6) of the cacao testa of three genotypes (CCN 51, ICS 95, TCS 01) fermented under three temperature profiles (T) for six days. Three sample units (14, 25, 36) per genotype are plotted.

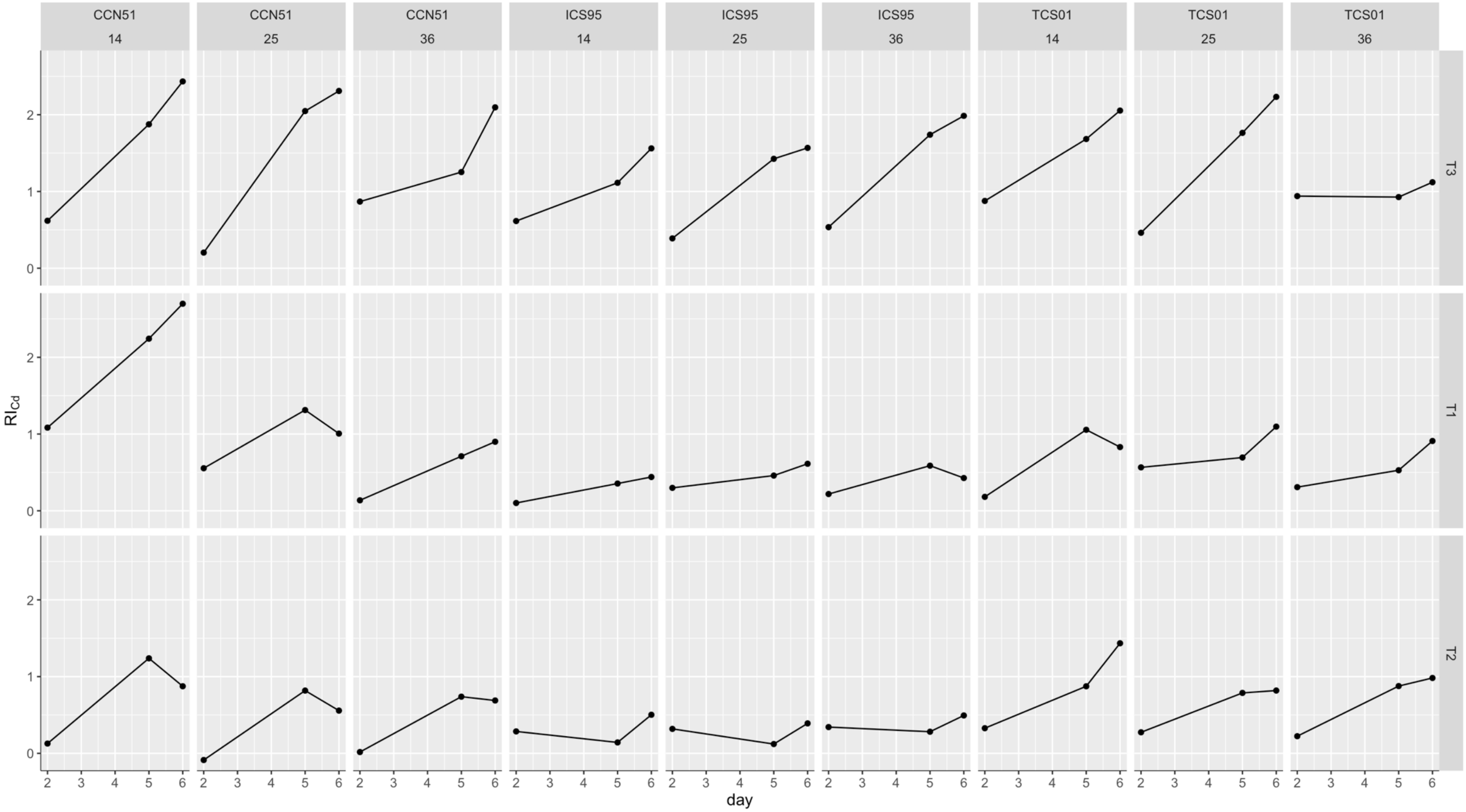

Figure S5. Time course for the internal translocation factor (ITF, estimated on days 2, 5, and 6) of cacao bean tissues of three genotypes (CCN 51, ICS 95, TCS 01) fermented under three temperature profiles (T) for six days. Three sample units (14, 25, 36) per genotype are plotted. An ITF higher than 1.0 indicates that testa Cd is higher than nib Cd concentration (horizontal reference line).

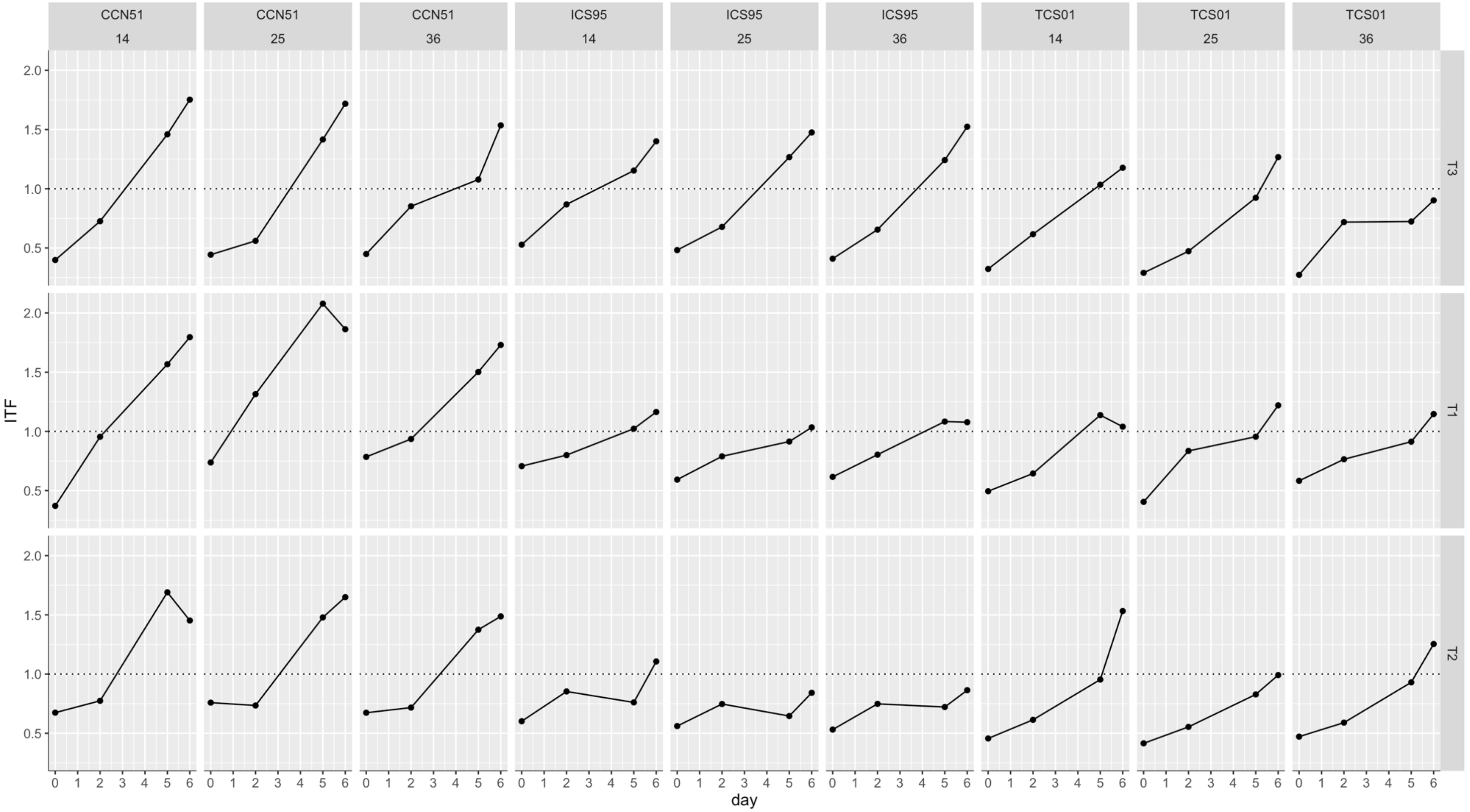

References

1. Rohan, T. A. Processing of raw cocoa. II.—Uniformity in heap fermentation and development of methods for rapid fermentation of west african amelonado cocoa. *Journal of the Science of Food and Agriculture* **9**, 542–551 (1958).
2. Forsyth, W. G. C. & Quesnel, V. C. The Mechanism of Cacao Curing. in *Advances in Enzymology and Related Areas of Molecular Biology* 457–492 (John Wiley & Sons, Ltd, 1963). doi:10.1002/9780470122709.ch10.
3. Lopez, A. S. & Dimick, P. S. Cocoa Fermentation. in *Biotechnology Set* 561–577 (John Wiley & Sons, Ltd, 2001). doi:10.1002/9783527620999.ch14j.
4. De Vuyst, L. & Weckx, S. The cocoa bean fermentation process: from ecosystem analysis to starter culture development. *Journal of Applied Microbiology* **121**, 5–17 (2016).
5. Herrera-Rocha, F. *et al.* Dissecting fine-flavor cocoa bean fermentation through metabolomics analysis to break down the current metabolic paradigm. *Sci Rep* **11**, 21904 (2021).
6. Howat, G. R., Powell, B. D. & Wood, G. a. R. Experiments on cocoa fermentation in West Africa. *Journal of the Science of Food and Agriculture* **8**, 65–72 (1957).
7. Rohan, T. A. Processing of raw cocoa. I.—Small-scale fermentation. *Journal of the Science of Food and Agriculture* **9**, 104–111 (1958).
